# Chromogranins can be measured in samples from cats and dogs

**DOI:** 10.1186/1756-0500-7-336

**Published:** 2014-06-04

**Authors:** Mats Stridsberg, Ann Pettersson, Ragnvi Hagman, Christoffer Westin, Odd Höglund

**Affiliations:** 1 Department of Medical Sciences, Uppsala University, SE-751 85 Uppsala, Sweden; 2 Department of Clinical Sciences, Swedish University of Agricultural Sciences, Box 7054, SE-750 07 Uppsala, Sweden

**Keywords:** Chromogranin, Secretogranin, Canine, Feline, Human, Radioimmunoassay

## Abstract

**Background:**

Methods for objective evaluation of stress in animals are important, but clinically difficult. An alternative method to study the sympathetic activity may be to investigate Chromogranin A (CGA), Chromogranin B (CGB) and Secretogranin II (SG2). The aim of this study was to investigate the cross-reactivity of CGA, CGB and SG2 between man, cat and dog and to explore possibilities to measure these proteins in samples from cats and dogs.

**Results:**

Adrenal gland extracts from feline and canine species were measured by region-specific radioimmunoassays in different dilution steps to explore possible inter species cross reactivity. High cross reactivity was found for cats in the CGA17-38, CGA324-337, CGA361-372, CGB and SG2 assays. High cross reactivity was found for dogs in the CGA17-38, CGA361-372, CGB and SN assays. The method measuring the intact CGA was not useful for measurements in cats and dogs.

**Conclusions:**

Region-specific assays measuring defined parts of CGA, CGB and SG2 can be used for measurements in samples from cats and dogs. These results are promising and will allow for further studies of these proteins as possible clinical biomarkers in cats and dogs.

## Background

Methods for objective evaluation of stress in animals are important, but clinically difficult. An alternative method to study the sympathetic activity may be to investigate Chromogranin A (CGA). CGA is part of a family of acidic and water soluble proteins called Granins, which also include Chromogranin B (CGB) and Secretogranins (SG) II-VII [1]. The Granins are stored in secretory granules of neuroendocrine tissues (chromaffin cells) and are released together with noradrenalin and adrenalin. Especially CGA is considered a reliable indicator of activation of the sympathetic tone [1,2]. Clinically, CGA and CGB are used for diagnosis and follow-up of various endocrine tumours [1]. CGA has also been proposed for evaluation of stress in intensive care units and for assessment of cardiovascular risk [3]. In critically ill patients an increased concentration of CGA is associated with shorter survival [4-8]. There are considerable differences in the amino acid composition between different animals and commercial assays for measuring human CGA can usually not be used for measuring CGA in samples from other species. However, some specific parts of the molecule have a higher degree of amino acid homology and methods where the antibodies are directed against specific epitopes can be used to measure samples from different animals [9].

The aim of this study was to investigate the cross-reactivity of CGA, CGB and SG2 between man, cat and dog and to explore possibilities to measure these proteins in samples from cats and dogs.

## Methods

### Collection of adrenals and extraction of Chromogranins

Adrenals were removed from a feline (Domestic short hair) and canine (German shepherd) specimen euthanized for reasons not associated with this study. The adrenals were weighed, frozen and lyophilized. The lyophilized material was homogenized in distilled water and boiled for 5 min. The mixture was centrifuged at 10 000 g for 15 min and the resulting supernatant was frozen in aliquots until measurements.

### Measurements of Chromogranins in adrenal extracts

Aliquots of supernatant derived from extracted adrenals were serial diluted in assay buffer and measured in the respective Granin assays. All assays were competitive radioimmunoassays, measuring defined parts of the Granin molecules. In this setting 5 region-specific assays were tested for determination of feline and canine CGA (CGA17-38, CGA176-195, CGA238-247, CGA324-337 and CGA361-372) [10]. The extracts were also tested in an assay measuring Secretoneurin (SN), which is a defined part of SG2 [11], and in two commercial radioimmunoassays measuring human CGA and CGB (Eurodiagnostica AB, Malmö Sweden). All assays had a total assay variation of <9%.

### Amino acid sequences for Chromogranins

Amino acid sequences for CGA (Accession numbers AAB53685. XP_006933167, XP_005623224), CGB (Accession numbers NP_001810, XP_006930003, XP_005634865) and SG2 (Accession numbers AAA36607, ABD24220, XP_545669) for human, feline and canine respectively, were obtained from the NCBI Protein data base.

### Statistics

Measured values were expressed as mean values, standard deviation and coefficient of variation (CV)% (=%SD/MV).

## Results

The results from the measurements of CGA, CGB and SN in the adrenal extracts from cats are shown in Table 1 and the results from the measurements in dogs are shown in Table 2. Different concentrations were obtained with the different region-specific assays for CGA and the other assays. Low CV indicates reproducible measurements in different dilutions, which is a reliable indicator of high cross-reactivity. High CV, on the other hand, indicates deviating results as signs of low cross-reactivity and these concentration results cannot be considered as true estimates. Since the total assay variation in all assays were <9%, a CV of <13.5% (=1.5xCV) was used as the criterion to accept an assay as suitable for measurements in the feline or canine species. Low CV was found for cats in the CGA17-38, the CGA324-337, CGA361-372, the CGB and the SN assays. Low CV was found for dogs in the CGA17-38, the CGA361-372, the CGB and the SN assays. The method measuring the whole intact CGA was not useful for measurements in cats and dogs.

**Table 1:** Assays used for the cross reactivity studies in adrenal extracts from cat

**Assay**	**MV**	**SD**	**CV**	**Suitable assay**	**Aa homology**
CGA 17-38	3,26	0,16	4,8%	Yes	100%
CGA 176-195	0,14	0,02	16,7%	No	59%
CGA 238-247	0,23	0,08	35,4%	No	90%
CGA 324-337	3,04	0,88	29,0%	No	93%
CGA 361-372	1,16	0,04	3,4%	Yes	83%
SG2 154-165	3,79	0,10	2,7%	Yes	100%
CGB (Eurodiagnostica)	2,31	0,30	12,8%	Yes	85%
CGA (Eurodiagnostica)	14,63	3,23	22,1%	No	

The table shows the assays used for the cross reactivity studies of Chromogranin A (CGA), Chromogranin B (CGB) and Secretogranin II (SG2) measurements in adrenal extracts from cat. A coefficient of variation (CV = SD/MV) less than 13.5% (=1.5 x assay CV) was considered acceptable for measurements i.e. determined as a suitable assay, see text for explanation. The amino acid (Aa) homology between the human and feline species is indicated for the different assays.

**Table 2:** Assays used for the cross reactivity studies in adrenal extracts from dog

**Assay**	**MV**	**SD**	**CV**	**Suitable assay**	**Aa homology**
CGA 17-38	3,02	0,26	8,8%	Yes	100%
CGA 176-195	0,10	0,02	15,5%	No	79%
CGA 238-247	0,24	0,11	47,0%	No	75%
CGA 324-337	1,41	0,18	12,7%	Yes	79%
CGA 361-372	0,46	0,02	3,6%	Yes	70%
SG2 154-165	7,22	0,48	6,7%	Yes	100%
CGB (Eurodiagnostica)	3,89	0,42	10,7%	Yes	77%
CGA (Eurodiagnostica)	4,99	1,22	24,5%	No	

The table shows the assays used for the cross reactivity studies of Chromogranin A (CGA), Chromogranin B (CGB) and Secretogranin II (SG2) measurements in adrenal extracts from dog. A coefficient of variation (CV = SD/MV) less than 13.5% (=1.5 x assay CV) was considered acceptable for measurements i.e. determined as a suitable assay, see text for explanation. The amino acid (Aa) homology between the human and canine species is indicated for the different assays.

The amino acid sequence homology between human and feline was highest for CGA17-38 (100%). Likewise, the homology was high, 100%, for SN, whereas the other assays had about 50-90% amino acid homology (Table 1). The amino acid sequence homology between human and canine was highest for CGA17-38 (100%) and the homology was high, 100%, also for SN, whereas the other assays had about 70-80% amino acid homology (Table 2).

## Discussion

The aim of this study was to investigate the cross-reactivity of CGA between man, cat and dog and to explore possibilities to measure CGA in samples from cats and dogs. Since plasma samples with high concentrations of CGA are difficult to find in animals, we choose to use extracts of Chromogranins from adrenal glands, an organ known to contain high concentrations of CGA, CGB and SG2 [12]. Using the supernatant from adrenal extracts, we were able to obtain high enough concentrations to allow serial dilution, which is an established way to explore cross reactivity. Similar experiments have been performed earlier for bovine, equine, ovine and caprine species [9]. In agreement with our results, as in the previous study [9], the CGA17-38 assay showed cross reactivity between the species, while the CGA assay measuring the whole intact CGA molecule, which corresponds to the Eurodiagnostica assay used in this study, was not useful. Interestingly, some assays with lower amino acid homology showed cross reactivity, while others did not. It is known that, in particular, charged amino acids are highly immunoreactive. An exchange between species of one or more of these crucial amino acids will give a high impact on the antibody binding to the antigen. It is thus possible that such substitution has occurred for the CGA176-195 and CGA238-247 parts of the molecule in cats and for the CGA176-195, CGA238-247 and CGA324-337 epitopes in dogs, thus explaining the poor cross reactivity although the apparent amino acid homology was rather high. Some CGA assays in the present study appear to be useful for measurements of Chromogranins in both cats and dogs. These assays measure known epitopes of CGA, which have been identified with biological activity (the CGA17-38 is also called Vasostatin and the CGA361-372 is also called Catestatin [13]). This is also the case with the part of SG2, *i.e*. SN, we have used in this study [11]. It is thus likely that peptides with biological activity have highly conserved inter-species amino acid homology and, as seen in this study, are useful for quantitative measurements.

All assays used in this study have previously been used for measurements of circulating concentrations of Chromogranins in plasma from human patients with neuroendocrine tumours [11,13]. However, Chromogranins have multiple sites that are likely to be cleaved both before and after release to the circulation. Accordingly different molecular forms of Chromogranins are likely to be found in the circulation. A more precise way to estimate the circulating concentrations of Chromogranins and other proteins, processing-independent assays, has been presented [14]. However, the use of region-specific assays with defined antibody epitopes, as in this study, can also be a way to assess the problem with different circulating molecular forms. It has also been shown that circulating concentrations of Chromogranins has a diurnal variation, a fact that should be considered in the planning of experiments [15].

CGA has been measured in plasma from cats and dogs with antibodies directed against both the C-terminal and the N-terminal of human CGA [16]. CGA has also been measured in plasma samples and adrenal extract from dogs [17,18]. Salivary concentrations have been measured in dogs [19]. This was done with an ELISA-kit from Yanaihara Institute, Japan, where the antibodies were directed against the epitope CGA344-374, which roughly covers the same region as one of the assays in this study, CGA361-372 (Catestatin).

## Conclusions

In conclusion, we have identified parts of CGA, CGB and SG2 and assays that can be used for measurements of samples from cats and dogs. These results are promising and will allow for further studies of these proteins as possible clinical biomarkers for sympathetic activity and stressful situations in cats and dogs and possible even other diseases.

### Ethics

Adrenals were removed from a feline and canine specimen euthanized for reasons not associated with this study. Tissue sampling was performed according to the ethical regulations in Sweden (Swedish Board of Agriculture, Dnr 38-9492/12) after written informed owner consent had been obtained.

## Abbreviations

CGA: Chromogranin A; CGB: Chromogranin B; SG2: Secretogranin II; SN: Secretoneurin.

## Competing interests

The authors declare that they have no competing interests.

## Authors’ contributions

MS planned the study, performed the laboratory work, performed the statistical analysis and drafted the manuscript. AP and RH participated in study design and coordination. CW conceived of the study and participated in study design. OH conceived of the study, participated in its design and coordination. All authors have participated in the preparation of the manuscript and approved the final manuscript.
